# Methicillin resistant *Staphylococcus aureus* isolated from surgical patients in Cambodia over a 10-year period

**DOI:** 10.1177/00494755231174261

**Published:** 2023-05-08

**Authors:** William Wong, Vi-Seth Bak, Weisang Luo, Suon Hoksear, Prum Rith, James Gollogly

**Affiliations:** 1 12205 Medical School, University of Oxford, Oxford, UK; 2 Adelaide Medical School, 1066 University of Adelaide, Adelaide, Australia; 3 Children's Surgical Centre, Phnom Penh, Cambodia

**Keywords:** Asia, surgery, bacterial infection, public health, musculoskeletal

## Abstract

Methicillin-resistant *Staphylococcus aureus* (MRSA) related surgical infections are a global challenge. The burden of antimicrobial resistance is high throughout South-East Asia, and this is reflected in our local institution in Cambodia. Between 2011 and 2013, we analysed 251 wound swab samples at the Children's Surgical Centre, Phnom Penh; 52.5% of the *Staphylococcus aureus* isolates (*n* = 52/99) were MRSA positive. Ten years on, we have sought to investigate whether there is a difference in MRSA rates within our adult and paediatric patient population. Between 2020 and 2022, MRSA rates in our patient population have remained similar at 53.8% (*n* = 42/78). Resistance profiles of MRSA isolates have also remained similar with a significant proportion of MRSA still showing sensitivity to trimethoprim-sulfamethoxazole and tetracycline. We also find that patients presenting with wound infection secondary to trauma or orthopaedic implants had greater propensity to yield MRSA.

## Introduction

Methicillin-resistant *Staphylococcus aureus* (MRSA) poses a serious threat to public health around the world due to its high level of mortality.^1–3^ It is an important cause of severe infections of the skin, soft tissues and bone, which may progress to sepsis. Infections by MRSA are prevalent across South-East Asia^4–6^ and recent reports have suggested that there may be an increasing trend of community-associated MRSA infections being detected within the region.^7^ The burden of antimicrobial resistance (AMR) is high in low- and middle-income regions of South-East Asia owing to antibiotic misuse and poor enforcement of antibiotic stewardship policies.^8^ Limited access to expensive diagnostic and treatment options only perpetuates the difficulties of managing antibiotic resistant infections.^9,10^ Cambodia, a country recovering from a 20th century genocide, has limited healthcare infrastructure and AMR stewardship. As such, the overall trends in the prevalence of MRSA within the country remain poorly understood.

The Children's Surgical Centre (CSC) is a charitable, non-governmental organisation (NGO) located in Phnom Penh. It serves as a national tertiary centre providing reconstructive and rehabilitative surgical treatment for patients of all age groups. The aim of the present study was to investigate whether MRSA rates at CSC have changed over a 10-year period.

## Methods

The CSC, Phnom Penh, triages between 10,000 and 14,000 patients per year who are either referred by other healthcare facilities or present independently without referral. Patients come from both the surrounding Phnom Penh area and other provinces across Cambodia for consultations, surgical treatment and rehabilitation. CSC consists of a team of local and international surgeons, nurses and physiotherapists who provide care to patients affected by traumatic injuries and congenital disabilities. The surgical specialties provided by CSC include orthopaedics, plastic and reconstruction, ear, nose and throat surgery, and ophthalmology. Whilst CSC performs approximately 4000 operations per year, it notably does not provide acute trauma surgery and many patients arrive at CSC already having received acute surgical care at other healthcare providers.

At CSC, wound swab cultures were collected as part of routine practice on the clinical discretion of the surgeons. Samples were placed in standard culture tubes containing bacterial transport media and sent with a corresponding specimen transport form to a microbiology laboratory in Cambodia. Swab cultures were taken from patients during the following study periods: Study Period 1 (October 2011 to April 2013) and Study Period 2 (January 2020 to December 2022). All samples from study period 1 were sent to Naval Medical Research Unit 2, Phnom Penh (NAMRU2-PP) laboratory. Owing to the closing of NAMRU2-PP in 2021, samples taken during study period 2 were also sent to National Reference Medical Laboratory (NRML), Phnom Penh and Amatak Medical Analysis Laboratory, Phnom Penh. Results of the biochemical analysis for species identification and antibiotic susceptibility testing were reported back to CSC by electronic mail.

Patients from whom wound swab cultures were collected were identified through CSC's paper and electronic medical records. Data collected from patient notes included patient demographics, type of sample, anatomical location, organism(s) isolated and their antibiotic susceptibility. We further categorised wound characteristics according to whether it was trauma related and/or had implanted orthopaedic metalwork. Presenting conditions were classified as being related to trauma if the initial condition was a burn, fracture or snakebite. Data were collected following ethical approval by the local institutional review board. This study was performed in accordance with the principles of the Declaration of Helsinki (last revised in 2013).

Statistical analyses were performed with Fisher exact tests using Chi-square contingency tables for the proportion of *S. aureus* infections that were MRSA for the following factors: gender of patient; trauma aetiology; presence of orthopaedic metalwork. A Kolgomorov-Smirnoff test was used to examine whether age distributions differed among patients infected by methicillin-sensitive *S. aureus* or MRSA.

## Results

Over both study periods, we collected 477 wound swab cultures from 421 patients; 311 (65.2%) cultures tested positive for bacterial isolates (Table 1). The proportion of wound swabs which tested positive for bacterial isolates was lower in study period 2 (59.7%) compared to study period 1 (70.1%)*.*

**Table 1.** Number of patients and wound swab cultures during study period 1 and 2.

	Overall	Study period 1 (October 2011 to April 2013)	Study period 2 (January 2020 to December 2022)
Number of patients	421	204	217
Number of wound swab cultures	477	251	226
Number of positive wound swab cultures (%)	311 (65.2%)	176 (70.1%)	135 (59.7%)

During both study periods, *Staphylococcus aureus* was the most common bacterium isolated. It represented 56.3% and 57.8% of all positive samples in study period 1 and 2, respectively. The seven most commonly isolated organisms were the same across both periods: *S. Aureus,* coagulase negative *Staphylococcus, Escherichia coli, Pseudomonas aeruginosa, Enterobacter cloacae, Proteus mirabilis* and *Klebsiella pneumoniae* (Table 2). Wound swabs which tested positive for *S. aureus* were taken from sites throughout the body; the lower and upper leg were the most common sites during both study periods.

**Table 2.** Most commonly isolated bacterial organisms during study period 1 and 2.

	Study period 1		Study period 2	
Organism	*N*	% of positive swab cultures	N	% of positive swab cultures
Staphylococcus aureus	99	56.3	78	57.8
Coagulase negative staphylococcus	36	20.5	8	5.9
Escherichia coli	36	20.5	12	8.9
Pseudomonas aeruginosa	24	13.6	21	15.6
Enterobacter cloacae	13	7.4	6	4.4
Proteus mirabilis	12	6.8	15	11.1
Klebsiella pneumoniae	10	5.7	12	8.9

As stated in our initial study,^11^ 52 of the 99 (52.5%) *S. aureus* isolates in study period 1 were MRSA. This was comparable to our more recent study period, with 42 out of 78 (53.8%) being MRSA (Table 3). The overall proportion of positive wound swab samples which grew MRSA was 29.5% (*n* = 52/176) and 31.1% (*n* = 42/135) in study periods 1 and 2, respectively.

**Table 3.** Number of wound swab cultures positive for *Staphylococcus aureus* and methicillin resistant *Staphylococcal aureus* (MRSA) during study period 1 and 2.

	Overall	Study period 1	Study period 2
Number of positive wound swab samples	311	176	135
Number of S. aureus positive samples	177	99	78
Number of MRSA positive samples (% of S. aureus positive samples)	94 (53.1%)	52 (52.5%)	42 (53.8%)

An analysis of the resistance profiles from our previous study had showed that all 52 wound swab cultures positive for MRSA were sensitive to vancomycin.^11^ In study period 2, sensitivity to vancomycin was only tested in 25 of 42 samples with MRSA. Of these, one was resistant to vancomycin. During study period 1, it was also observed that a portion of the MRSA isolates retained their susceptibility to certain antibiotics, namely trimethoprim-sulfamethoxazole (61.5%; *n* = 32/52) and tetracycline (51.9%; *n* = 27/52)^.^^11^ A similar trend was also evident during study period 2, with sensitivity to trimethoprim-sulfamethoxazole being observed in 34 out of 42 isolates (81.0%), and sensitivity to tetracycline being detected in 25 out of 44 isolates (56.8%).

The 177 wound swab cultures which grew *S. aureus* across both study periods came from 162 patients; 114 patients were male and 48 were female (Table 4). 82.4% (*n* = 70/85) of the patients with MRSA infections were male compared with only 57.1% (*n* = 44/77) of patients with sensitive *S. aureus*. There was a statistically significant relationship between gender and the type of *S. aureus* infection, with males being more likely to have MRSA infections compared to females (2 ×  2 contingency table, Fisher's exact test, two tailed, *p* = 0.0005). The mean age of patients in study period 1 and 2 was 31.5 and 31.8, respectively (Table 4). There was no difference between the cumulative age distributions of patients infected with MRSA versus methicillin sensitive *S. aureus* (Kolgomrov-Smirnoff Test, D = 0.13, *p* = 0.48).

**Table 4.** Patient characteristics of *staphylococcal aureus* positive patients.

	Overall	Study period 1	Study period 2
Number of patients with S. Aureus	162	89	73
Males:Females	114:48	59:30	55:18
Mean age (Range)	31.6 (2–76)	31.5 (2–76)	31.8 (2–71)

Information regarding presenting aetiology and the presence of orthopaedic metalwork was available for 159 *S. aureus* positive patients across both study periods. The majority of these patients (77.4%, *n* = 123/159) presented to the centre due to a trauma-related aetiology. Among the trauma-related cases, 58.5% (*n* = 72/123) grew MRSA, while only 30.6% (*n* = 11/36) of non-trauma-related cases grew MRSA. This suggests that patients who attended the centre due to trauma-related presentations were more likely to grow MRSA than those who presented for non-traumatic reasons (2 × 2 contingency table, Fisher's exact test, two tailed, *p* = 0.004). Of the *S. aureus* patients with available information, most had existing orthopaedic metalwork upon presentation to CSC (52.2%, *n* = 83/159). Further analysis revealed that 67.5% (*n* = 56/83) of wounds associated with orthopaedic metalwork grew MRSA, while 36.8% (*n* = 28/76) of wounds without metalwork grew MRSA. This difference in proportions was found to be significant (2 × 2 contingency table, Fisher's exact test, two-tailed, *p* = 0.0001).

## Discussion

Our study aimed to assess whether the bacterial profile of wound infections amongst patients at the CSC in Phnom Penh, Cambodia, had changed over a 10-year period. Our findings suggest that MRSA continues to be a significant problem in wounds infected by *S. aureus* amongst the adult and paediatric patients who come to the Children's Surgical Centre. To our knowledge, there has only been one other study of MRSA rates in wound infections in Cambodia.^12,13^ This study, which examined the bacterial profile of post caesarean surgical site infections in the Kampong Cham province, reported a similar MRSA rate of 50%.^13^ However, it is important to note that the sample of *S. aureus* positive patients in this study from 2013 was much smaller (*n* = 2). Without additional studies of the bacterial profile of wound infections from across Cambodia, it will be challenging to establish whether the high rate of MRSA reported at the Children's Surgical Centre is also seen across the whole country. Nevertheless, the results of our study provide an important snapshot of MRSA rates within Phnom Penh and the surrounding provinces from which many our patients present.

Cambodia's limited healthcare infrastructure and AMR stewardship make it difficult to understand the trends in MRSA within the country. The first attempt to isolate MRSA in Cambodia was described in a small surveillance study within the Battambang province in 2002, which found no evidence of MRSA in nine patients with *S. aureus-*positive cultures.^14^ MRSA was subsequently first isolated in the Siem Reap province in 2006^15^ and was determined to exist at a 3.5% carrier rate within their paediatric population.^16^ In this study, we found that MRSA rates have remained stable at the CSC between 2011 and 2022. This is in line with findings from the only other investigation into recent MRSA trends in Cambodia, which also reported stable MRSA rates between 2007 and 2016 in their paediatric patient population.^17^ However, unlike our study which reported an overall 53.1% MRSA rate across the combined study period, the study conducted by the Angkor Hospital for Children only reported a 13.0% MRSA rate. This difference could potentially be attributed to variations in the patient population and sample types between our two studies. Specifically, our study analysed wound infections in both adults and children, while their study only focused on blood and cerebrospinal fluid samples from children. It is also important to note that many of the patients presenting to the CSC had previously suffered traumatic injuries and received treatment, such as antibiotics and surgery, at other healthcare facilities within Cambodia. A significant proportion of these patients also choose to receive treatment in the community, including Khmer traditional medicine, prior to attending CSC.

Our study found that patients who presented to CSC with trauma-related causes or implanted orthopaedic metalwork were more likely to grow MRSA in their wounds. In our study, 58.5% (*n* = 72/123) of trauma related cases and 67.5% (*n* = 56/83) of patients with implanted orthopaedic metalwork grew MRSA. In comparison, only 30.6% (*n* = 11/36) of non-trauma-related cases and 36.8% (*n* = 28/76) of patients without metalwork grew MRSA. These findings could potentially be attributed to factors related to the management of patients with traumatic injuries such as prolonged hospitalisation or antibiotic treatment, which increase the risk of growing antibiotic-resistant bacterial infections. Patients with orthopaedic metalwork or open traumatic wounds are also at higher risk of acquiring MRSA in the hospital environment where it is commonly found. Our study was limited in that we were unable to collect information on the types of medical and surgical treatments patients received at other healthcare facilities or within the community prior to arriving at CSC. Furthermore, we were unable to ascertain whether patients had previously grown MRSA in cultures performed at other healthcare centres. This limits our ability to determine whether patients may have acquired MRSA from CSC, other healthcare facilities, or from the community. While the misuse of antibiotics in the community is well documented in Cambodia^18,19^ there is little information on how antibiotics are used in trauma settings in healthcare facilities in Cambodia, specifically in the pre-, peri-, and post-operative periods. As Cambodia continues to develop and the number of surgical care providers increases, there is a need to investigate further and regulate the usage of antibiotics in these facilities to prevent the spread of antibiotic-resistant infections such as MRSA.

In most countries across the world, vancomycin remains the first-line treatment for MRSA infections; as such, the increasing reports of vancomycin resistant SA (VRSA) is of great concern.^20^ In our previous study we had shown that vancomycin was effective against all MRSA isolates tested from CSC (*n* = 52/52). Ten years on, we have identified one instance of vancomycin resistance within 25 samples positive for MRSA. Although vancomycin has gradually become more accessible in Cambodia since our original study, whether this is driving an increase in the rates of VRSA will require further study across a larger patient population. Importantly, our recent analysis also showed that many of the cases of MRSA within our population have remained sensitive to trimethoprim-sulfamethoxazole (‘bactrim’), a low-cost antibiotic treatment which is still much more accessible across Cambodia compared to vancomycin.

Further studies will be needed to identify effective strategies for the prevention and management of MRSA infections in surgical patients within Cambodia. Our findings also highlight the need for continued monitoring and analysis of MRSA rates in Cambodia as the country works to address AMR through sustained efforts with the World Health Organisation.^21,22^
